# Building on the success of anti-vascular endothelial growth factor therapy: a vision for the next decade

**DOI:** 10.1038/s41433-020-0895-z

**Published:** 2020-06-15

**Authors:** Anthony P. Adamis, Christopher J. Brittain, Atul Dandekar, J. Jill Hopkins

**Affiliations:** Genentech, Inc., South San Francisco, CA USA

**Keywords:** Retinal diseases, Quality of life

## Abstract

This article aims to identify key opportunities for improvement in the diagnosis and treatment of retinal disease, and describe recent innovations that will potentially facilitate improved outcomes with existing intravitreal vascular endothelial growth factor (VEGF) therapies and lay the groundwork for new treatment approaches. The review begins with a summary of the key discoveries that led to the development of anti-VEGF therapies and briefly reviews their impact on clinical practice. Opportunities for improvements in diagnosis, real-world outcomes with existing therapies, long-acting therapeutics and personalised health care are discussed, as well as the need to identify new targets for therapeutic intervention. Low-cost, remote patient screening and monitoring using artificial intelligence (AI)-based technologies can help improve diagnosis rates and enable remote disease monitoring with minimal patient burden. AI-based tools can be applied to generate patient-level prognostic data and predict individual treatment needs, reducing the time needed to optimise a patient’s treatment regimen. Long-acting therapeutics can help improve visual outcomes by reducing the treatment burden. When paired with AI-generated prognoses, long-acting therapeutics enable the possibility of vision loss prevention. Dual-acting drugs may help improve efficacy and/or durability beyond what is possible with anti-VEGF agents alone. Recent developments and ongoing innovations will help build upon the success of anti-VEGF therapies to further reduce vision loss owing to retinal disease while lowering the overall burden of care.

## 摘要

本文旨在挖掘提高视网膜疾病诊断和治疗方面的契机, 描述了近年来可能有助于改善现有玻璃体腔内注射血管内皮生长因子 (vascular endothelial growth factor, VEGF) 治疗结局的新思路, 以及它们如何为今后新的治疗奠定基础。本综述首先概述了促使抗VEGF治疗实现临床转化过程中一些关键性发现, 并简要回顾了它们对临床实践的影响。本文也讨论了如何提高诊断、现有治疗的真实世界结局、长效以及个体化治疗策略, 以及确定治疗干预目标的必要性。利用人工智能 (artificial intelligence, AI) 技术的低成本、远程患者筛查和监测功能有助于提高诊断效率, 以最小的患者负担成本实现远程疾病监测。AI设备可提供患者的疾病预后数据以及预测个体治疗的需求, 减少优化患者治疗方案所需的时间。长效治疗通过减轻治疗负担改善视力结局。长效治疗与AI提供的患者预后相结合可预防视力丧失。与抗VEGF单药治疗相较, 联合治疗药物有助于提高疗效和/或持久性。近年来的不断发展以及创新将有助于在抗VEGF治疗成功的基础上, 进一步减少视网膜疾病所致的视力损失, 同时降低总体医疗负担。

## Introduction

The approval of the first vascular endothelial growth factor (VEGF) inhibitor for neovascular age-related macular degeneration (nAMD) in 2004 heralded in a new era in the treatment of retinal disease. Before the introduction of anti-VEGF drugs, the best treatments available for disorders such as nAMD and diabetic macular oedema (DMO) could only slow the rate of vision loss [1, 2]. Anti-VEGF therapy provided the first opportunity to restore functionally meaningful vision in patients with these conditions [3, 4], transforming patients’ lives and shifting the goal of treatment from sight preservation alone to sight restoration.

Although anti-VEGF agents have reduced the rates of blindness from nAMD worldwide [5, 6] and will likely have a similar impact on blindness owing to DMO/diabetic retinopathy (DR) [7], these diseases continue to be leading causes of visual impairment [8] and the resultant societal costs remain high [9]. We believe there are several opportunities for improved outcomes in nAMD and DMO/DR. The aims of this paper are to (1) briefly review the history of intravitreal anti-VEGF therapies, and (2) highlight ways we can build upon their success. By leveraging advancements in artificial intelligence (AI), personalised health care, long-acting therapeutics and novel drug targets, we believe it is possible to minimise vision loss and achieve better overall management of these diseases over the next decade.

## Development of intravitreal VEGF inhibitors

Beginning in 1948, investigators hypothesised that a diffusible chemical factor emanating from the retina guided the development of the retinal vasculature [10]. This ‘Factor X,’ as it was termed by George Wise, appeared to be ‘intimately associated with the hypoxic retina,’ and it was hypothesised to be operative in multiple adult ischaemic retinopathies [11]. The 50-year search for Factor X ended with the discovery of VEGF [12] and the elucidation of its central role in DR [13], DMO [4] and retinal vein occlusion [14]. VEGF was also shown to have a pivotal role in nonischaemic retinal diseases such as nAMD [3, 15] and myopic choroidal neovascularisation [16].

The first anti-VEGF agent for use in the eye, pegaptanib (Macugen, Eyetech, Inc., New York, NY), was approved in 2004. Pegaptanib binds to a subset of VEGF-A isoforms and reduced the average number of letters lost in nAMD by 50% at 54 weeks [17]. Unlike the standard of care at the time (verteporfin; Visudyne, Bausch and Lomb, Inc., Bridgewater, NJ), pegaptanib was effective in all forms of nAMD [17].

Ranibizumab (Lucentis, Genentech, Inc., South San Francisco, CA) was the first anti-VEGF agent to neutralise all VEGF-A isoforms. It was also the first treatment of any kind to restore functionally meaningful vision in many patients with nAMD and to dramatically reduce the risk of progression to blindness [3, 18]. In a randomised controlled trial in nAMD [3], the percentage of patients with 20/40 or better vision improved from 4% at baseline to 39% after 12 months of monthly ranibizumab 0.5 mg, while remaining below 3% in eyes treated with verteporfin [3]. The rate of legal blindness (20/200 or worse) in the affected eye decreased from 23% at baseline to 16% at month 12 with monthly ranibizumab 0.5 mg, while nearly doubling in the verteporfin group (from 32% to 60%) [3]. Ranibizumab subsequently became the first anti-VEGF agent approved for the treatment of DMO [4], the leading cause of vision loss in DR, and then for DR itself [16]. In eyes with vision loss due to DMO, the percentage of patients with 20/40 or better vision in the two pivotal studies improved from 14–19% at baseline to 54–60% after 12 months of monthly ranibizumab 0.3 mg [4]. Improvements in vision were accompanied by significant improvements in DR severity, and many eyes with proliferative DR reverted to nonproliferative DR, a less sight-threatening form of the disease [16]. Ranibizumab was also shown to be effective in myopic choroidal neovascularisation, with >37% of ranibizumab-treated patients gaining ≥15 letters after 3 months versus <15% of photodynamic therapy-treated patients [16].

Aflibercept (Eylea, Regeneron Pharmaceuticals, Inc., Tarrytown, NY) was developed after ranibizumab and inhibits VEGF-A and placental growth factor [19]. Aflibercept produces visual outcomes comparable with ranibizumab in nAMD [19], but the recommended dosing is once every 8 weeks (after three to five monthly loading doses) for nAMD and DMO/DR [19]. Brolucizumab (Beovu, Novartis Pharmaceuticals Corporation, East Hanover, NJ) also was recently approved for nAMD in the United States, demonstrating noninferior visual outcomes compared with aflibercept.

Bevacizumab is an anti-VEGF agent first approved for use in colon cancer in 2004. It is approved for use in a variety of solid tumours, but not for use in the eye [20]. Although marginally less effective than ranibizumab and aflibercept in DMO [21], it is commonly used off label for retinal and choroidal vascular disease.

## Impact of anti-VEGF therapies and opportunities for improvement

Real-world studies in several countries have shown that the adoption of intravitreal anti-VEGF therapy as the standard of care has resulted in a decrease of ~50% in the number of people becoming legally blind owing to nAMD [5, 6]. It is also estimated that anti-VEGF therapy could decrease the 2-year rate of visual impairment or legal blindness due to DMO by 45% and 75%, respectively [7]. An epidemiological study in Ireland found that the rate of blindness owing to DR decreased by ~50% from 2011 (30.9 per 100,000 individuals with diabetes) to 2013 (14.9 per 100,000 individuals with diabetes), although the rate of visual impairment owing to DR remained fairly steady (12.3–11.7 per 100,000 individuals with diabetes), highlighting the importance of screening programmes for diabetic eye disease [22].

Despite the impressive impact of anti-VEGF therapies, real-world data indicate that millions of people with nAMD do not achieve the outcomes seen in registrational trials and are still suffering from moderate-to-severe vision impairment or blindness [8].

It is important to understand the root causes of the vision loss that persists and to propose strategies to reduce it. The sections that follow are devoted to this task.

### Problem statement: underdiagnosis

It is estimated that up to half of all patients with DR [23] and up to 25% of patients with nAMD [24] may be undiagnosed, depriving them of early treatment and increasing their risk of irreversible vision loss [16, 25]. Contributing factors likely include the fact that many people do not visit a doctor regularly, and that the cost of community-based screening programmes using existing methodologies can be high. Although current screening systems in some countries are effective, they are often limited to DMO/DR or glaucoma, and do not cover other retinal diseases such as nAMD.

#### Potential solution: AI-enabled, low-cost, remote screening

One potential solution is to use accurate and easy-to-use diagnostic tools to quickly and economically screen large numbers of individuals outside of physicians’ offices for any retinal disease. Recent advances in AI and cloud computing may make this possible.

Deep learning (DL; a type of AI) uses a computer-based neural network that can train itself on a large database to detect an outcome of interest. This approach is being increasingly used in health care to aid diagnosis and gain insights into disease processes [26]. DL is most advanced in the realm of computer vision (evaluation of images). The algorithms can evaluate minute details of millions of high-resolution medical images to accurately diagnose diseases and predict outcomes. Algorithms are more consistent than human experts and may recognise features that are not detectable by human evaluators and/or that had not been suspected of being related to the outcome. This can both increase diagnostic accuracy and provide insights into previously unidentified pathophysiological processes [26]. For example, a recent paper demonstrated that an imaging algorithm could accurately predict age, sex, smoking status and systolic blood pressure from a colour fundus photograph [27], something humans cannot do. In retinal disease, the vast databases of optical coherence tomography (OCT) images and fundus photographs, together with longitudinal data on visual function and treatments, provide a rich resource for training these algorithms.

In April 2018, the US Food and Drug Administration approved the first cloud-based DL algorithm paired with an autonomous retinal fundus camera to automatically identify eyes with DR requiring referral to an ophthalmologist [28]. Using a publicly available set of fundus images and a consensus reference standard set by three US board-certified retinal specialists, the algorithm had a sensitivity of 96.8%, a specificity of 87.0% and only six out of 874 false negatives [28]. Taking this concept one step further, Arcadu et al. [26] recently built a DL algorithm that predicted OCT measures of DMO from two-dimensional colour fundus photographs. Once validated, this algorithm, paired with low-cost digital fundus image capture and cloud computing, could serve as the basis for widespread decentralised screening programmes for DMO, potentially facilitating the timely triage of patients with sight-threatening disease to the care they need to preserve their sight.

### Problem statement: suboptimal outcomes in the real world

Several studies have shown that real-world outcomes with anti-VEGF therapy invariably fall short of those seen in phase 3 anti-VEGF clinical trials; these poorer outcomes are often associated with less frequent treatment [29, 30]. The need for frequent patient monitoring and intravitreal injections can place a high burden on patients, their caregivers and health care providers [31, 32], and this could be affecting outcomes in clinical practice.

#### Potential solution: long-acting therapeutics

One way to relieve the burden of frequent patient monitoring and treatment would be with effective long-acting anti-VEGF therapeutics. Several strategies are actively being investigated, including slow-clearing large molecules, slow-release formulations, continuous delivery drug-device combination technologies and gene therapy [33, 34]. The majority of these technologies are currently in preclinical or early clinical development. However, the Port Delivery System with ranibizumab (PDS) is currently being evaluated in a phase 3 clinical trial [33]. The PDS is a permanent, refillable intravitreal implant for the delivery of ranibizumab for extended durations. In the phase 2 trial, the PDS filled with ranibizumab 100 mg/mL achieved visual outcomes comparable with monthly ranibizumab injections but with a reduced treatment burden; ~80% of patients did not require a refill for ≥6 months [33]. The median time to first refill was 15 months [33]. Another approach recently approved by the US Food and Drug Administration is the injection of a significantly greater molar concentration of a VEGF inhibitor (brolucizumab).

### Problem statement: patient heterogeneity with respect to treatment needs

The HARBOR [35] and CATT [36] clinical trials demonstrated that the need for anti-VEGF injections varies widely between patients with nAMD. Some patients achieved disease quiescence after a few injections, while others required monthly injections for ≥1 year and still showed signs of disease activity [35]. The majority of patients required between seven to eight injections per year. Treatment needs also can change with time within the same patient [21]. These phenomena underlie the need for frequent office visits. To date, available data sets and traditional statistical methods have been unable to identify factors that could reliably predict treatment need and/or response at the patient level. For both existing and long-acting anti-VEGF therapeutics, the ability to predict the optimal treatment frequency at baseline and/or identify disease reactivation remotely will be key to ensuring the best possible treatment outcomes.

#### Potential solution: remote disease monitoring

The current standard of care involves frequent best-corrected visual acuity and OCT monitoring of disease activity in a retinal specialist’s office. This is a significant burden for patients, caregivers, health care providers and the health system, especially considering that most patients do not require treatment at every visit. However, if the visit schedule is not frequent enough, detection of disease reactivation may be late and vision may be permanently lost owing to delay in treatment. One potential solution is an accurate, economical and user-friendly remote monitoring system; efforts are underway using remote vision testing [37, 38] and remote low-cost OCT. Because remote assessments are conducted more frequently, the signal-to-noise ratio will likely increase, potentially raising the sensitivity and specificity of this approach. One home vision monitoring system recently demonstrated that daily testing can result in early nAMD detection and significantly reduce the risk of vision loss [39].

#### Potential solution: AI-assisted treatment frequency predictions

Bogunovic et al. [40] recently demonstrated that patients requiring ≥16 injections over 2 years (‘high-needs’ patients) could potentially be identified using a DL algorithm. If further refined and validated, this algorithm could allow high-needs patients to be placed on long-acting therapy early, avoiding the burden of multiple frequent evaluations and injections required to determine the patient’s treatment needs empirically, and reducing the risk of vision loss in patients who cannot maintain a frequent treatment schedule. Similarly, a DL algorithm could identify low-needs patients who might benefit most from remote monitoring [40], sparing these patients from frequent office visits and greatly reducing the overall burden and cost of care. Using this approach to deliver personalised care will help us move toward optimal vision outcomes with anti-VEGF therapy.

### Problem statement: the anti-VEGF-A efficacy ceiling

Multiple trials have demonstrated that anti-VEGF therapy has reached an efficacy ceiling; specifically, that visual acuity cannot be further improved with greater anti-VEGF-A potency and/or higher doses. For example, in the HARBOR trial, there was no improvement in visual outcomes in patients with nAMD when the ranibizumab dose was increased from 0.5 mg to 2.0 mg [35]. A similar ceiling effect has been seen with other potent anti-VEGF agents in nAMD (e.g., aflibercept, brolucizumab). Peak vision gains are similar across all currently approved agents, with no improvement in outcomes with increasing dose [41, 42]. As a result, several therapies that target VEGF plus a second pathophysiological mechanism are currently in clinical development.

#### Potential solution: targeting VEGF-A plus a second mechanism

Since the introduction of anti-VEGF agents for retinal disease ~15 years ago, no drug targeting a different pathophysiological mechanism has received regulatory approval. The one that is currently at the farthest point in clinical development (phase 3 testing) is the VEGF–angiopoietin (Ang)-2 bispecific antibody, faricimab. Like VEGF, the Ang-2–Tie2 growth factor receptor pathway is operative in normal retinal vascular development [43] and the increased vascular permeability and inflammation seen in DR [44]. In brief, in the normal retina, Ang-1 binding to the Tie2 receptor promotes vascular stability. In the diseased retina, there is a switch to Ang-2 production, promoting destabilisation of the vasculature, subclinical inflammation, vascular leakage and/or neovascularisation [45]. In a phase 2 clinical trial in DMO, simultaneously targeting both VEGF and Ang-2 with faricimab resulted in significantly greater improvements in best-corrected visual acuity, OCT thickness, DR severity and durability compared with ranibizumab 0.3 mg [46]. Phase 2 clinical trials in nAMD demonstrated that visual outcomes with faricimab every 12–16 weeks were similar to those with monthly ranibizumab 0.5 mg [47]. Both vision and durability endpoints are being evaluated in the phase 3 programme.

### Problem statement: it is not clear who should receive an anti-VEGF monotherapy vs a dual-targeted drug

If a new dual mechanism of action drug with superior efficacy and/or durability becomes available, it may not be apparent at first diagnosis whether it should be used first or second line. Many patients do well with anti-VEGF monotherapy, achieving good visual outcomes with relatively infrequent treatment. Conversely, some patients still exhibit signs of disease activity and have poor visual function despite frequent injections (i.e., incomplete responders). These patients might have better outcomes if treated with a bispecific antibody as a first-line therapy. Unfortunately, it is not currently possible to identify incomplete responders a priori.

#### Potential solution: DL algorithms to predict treatment response

DL proof-of-concept algorithms have been developed that use early functional and anatomic data to predict the treatment response following 12 months of monthly anti-VEGF therapy [48]. If these algorithms can be further improved and validated, it may be possible to identify ‘incomplete responders’ who would benefit from receiving a bispecific therapy first line. This will allow patients to receive the effective therapy they need without delay and help reduce their risk of irreversible vision loss. Conversely, such an algorithm could help identify patients who will do well with traditional anti-VEGF monotherapy.

### Problem statement: many patients present with irreversible vision loss

Underdiagnosis, delayed diagnosis and delays in treatment can all lead to irreversible vision loss. Remote screening for active disease can help, but likely will not capture all patients before the onset of irreversible vision loss.

#### Potential solution: use AI to identify high-risk patients with good vision and treat before vision loss

A DL algorithm has been created that predicts second eye conversions from intermediate AMD to clinically significant nAMD within 12 months [49]. Once refined and validated, this algorithm could be used together with long-acting therapeutics to prevent patients who already have nAMD in one eye from developing bilateral nAMD. Preclinical and clinical studies have shown that continuous anti-VEGF monotherapy can prevent the growth of new [15] and existing choroidal neovascularisation [3, 18]. If eyes with high-risk intermediate AMD can be accurately identified using AI-based tools, the use of safe and minimally invasive long-acting anti-VEGF therapeutics may help prevent the conversion to vision-threatening nAMD.

## Conclusions

**Fig. 1:**
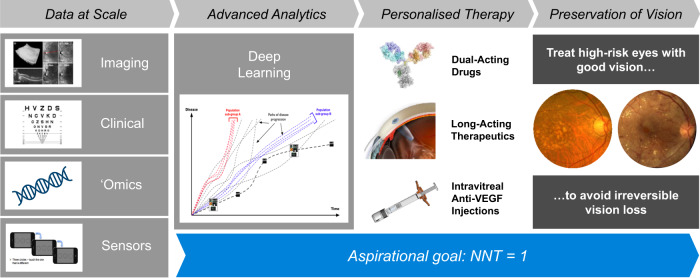
Use of data and advanced analytics to improve outcomes. Over the next decade, large multidimensional data sets, together with advanced deep learning algorithms, will facilitate the optimal treatment of patients. In addition, prophylaxis of high-risk eyes may prevent vision loss before any occurs. NNT indicates number of patients that need to receive treatment in order for one patient (on average) to derive benefit; VEGF, vascular endothelial growth factor.

In the 15 years since the development of the first intravitreal anti-VEGF therapy, there has been a transformation in the way we treat retinal and choroidal disease. With new tools and platforms such as AI-assisted remote screening and monitoring, predictive algorithms that allow personalised treatment strategies, long-acting therapeutics and new dual-targeted drugs, it may be possible to move closer to a goal of zero blindness due to nAMD and DR in the next decade (Fig. 1). It is hoped that the personalisation of therapy afforded by accurate AI and remote monitoring will eventually allow the field to approach the coveted ‘number needed to treat’ of 1, requiring only a single patient to be treated for every single case of vision loss prevented. This will allow every patient to be assured that they will receive the best treatment plan, at the right time, while also lowering the treatment burden for all involved. This review provides a potential roadmap to be followed in the attempt to achieve these goals.
